# Midtrimester preterm prelabour rupture of membranes (PPROM): expectant management or amnioinfusion for improving perinatal outcomes (PPROMEXIL – III trial)

**DOI:** 10.1186/1471-2393-14-128

**Published:** 2014-04-04

**Authors:** Augustinus S P van Teeffelen, David P van der Ham, Christine Willekes, Salwan Al Nasiry, Jan G Nijhuis, Sander van Kuijk, Ewoud Schuyt, Twan L M Mulder, Maureen T M Franssen, Dick Oepkes, Fenna A R Jansen, Mallory D Woiski, Mireille N Bekker, Caroline J Bax, Martina M Porath, Monique W M de Laat, Ben W Mol, Eva Pajkrt

**Affiliations:** 1 Department of Obstetrics and Gynaecology, GROW – School for Oncology and Developmental Biology, Maastricht University Medical Centre, P. Debijelaan 25, 6229 HX Maastricht, The Netherlands; 2 Department of Obstetrics and Gynaecology, Martini hospital, Groningen, The Netherlands; 3 Department of Obstetrics and Gynaecology, Academic Medical Centre, Amsterdam, The Netherlands; 4 Department of Epidemiology, Maastricht University Medical Centre, Maastricht, The Netherlands; 5 Julius Centre for Health Sciences and Primary Care, University Medical Centre, Utrecht, The Netherlands; 6 Department of Neonatology, Maastricht University Medical Centre, Maastricht, The Netherlands; 7 Department of Obstetrics and Gynaecology, University Medical Centre Groningen, Groningen, The Netherlands; 8 Department of Obstetrics, Leiden University Medical Centre, Leiden, The Netherlands; 9 Department of Obstetrics and Gynaecology, University Medical Centre Nijmegen, Nijmegen, The Netherlands; 10 Department of Obstetrics and Gynaecology, VU University Medical Centre, Amsterdam, The Netherlands; 11 Department of Obstetrics and Gynaecology, Maxima Medical Centre, Veldhoven, The Netherlands; 12 School of Paediatrics and Reproductive Health, The Robinson Institute, University of Adelaide, Adelaide, Australia

**Keywords:** PPROM, Oligohydramnios, Amnioinfusion, Perinatal mortality, Pulmonary hypoplasia

## Abstract

**Background:**

Babies born after midtrimester preterm prelabour rupture of membranes (PPROM) are at risk to develop neonatal pulmonary hypoplasia. Perinatal mortality and morbidity after this complication is high. Oligohydramnios in the midtrimester following PPROM is considered to cause a delay in lung development. Repeated transabdominal amnioinfusion with the objective to alleviate oligohydramnios might prevent this complication and might improve neonatal outcome.

**Methods/Design:**

Women with PPROM and persisting oligohydramnios between 16 and 24 weeks gestational age will be asked to participate in a multi-centre randomised controlled trial. Intervention: random allocation to (repeated) abdominal amnioinfusion (intervention) or expectant management (control). The primary outcome is perinatal mortality. Secondary outcomes are lethal pulmonary hypoplasia, non-lethal pulmonary hypoplasia, survival till discharge from NICU, neonatal mortality, chronic lung disease (CLD), number of days ventilatory support, necrotizing enterocolitis (NEC), periventricular leucomalacia (PVL) more than grade I, severe intraventricular hemorrhage (IVH) more than grade II, proven neonatal sepsis, gestational age at delivery, time to delivery, indication for delivery, successful amnioinfusion, placental abruption, cord prolapse, chorioamnionitis, fetal trauma due to puncture. The study will be evaluated according to intention to treat. To show a decrease in perinatal mortality from 70% to 35%, we need to randomise two groups of 28 women (two sided test, β-error 0.2 and α-error 0.05).

**Discussion:**

This study will answer the question if (repeated) abdominal amnioinfusion after midtrimester PPROM with associated oligohydramnios improves perinatal survival and prevents pulmonary hypoplasia and other neonatal morbidities. Moreover, it will assess the risks associated with this procedure.

**Trial registration:**

NTR3492 Dutch Trial Register (http://www.trialregister.nl).

## Background

Preterm prelabour rupture of membranes (PPROM) before or near the limit of viability is associated with high perinatal morbidity and mortality. Respiratory complications are frequent after periviable PPROM, as well as sepsis, intraventricular haemorrhage, retinopathy and necrotising enterocolitis (NEC). Among respiratory complications pulmonary hypoplasia is an important cause of death. Other respiratory complications consist of pneumonia, Infant respiratory distress syndrome (IRDS) and bronchopulmonary dysplasia (BPD).

PPROM before 26 weeks can delay lung development and can cause pulmonary hypoplasia [1]. Pulmonary hypoplasia is a term to describe an altered pulmonary development characterised by a reduction in the number of pulmonary alveoli or in bronchial branching. In fetal lung development a critical interval, the canalicular phase, exists between 16 and 28 weeks gestation. Gestational age at rupture of membranes has been shown to be inversely related to the risk of pulmonary hypoplasia.

Pulmonary hypoplasia results in severe respiratory failure leading to early neonatal death, respiratory insufficiency with pulmonary haemorrhage, bronchopulmonary dysplasia, or sub-acute lung disease, or sometimes in mild transient respiratory disease [2]. Perinatal mortality approximates 70% in most series (55-100%) [3].

In a review of 11 studies on midtrimester PPROM, the reported incidence of pulmonary hypoplasia secondary to midtrimester PPROM ranged widely from 1% to 48% [4]. A review of 6 studies on PPROM before 24 weeks reported an incidence of pulmonary hypoplasia of 19% (n = 120) [5]. These percentages do not represent the true natural history, given the limitations of these reviews, summarising only retrospective cohorts from tertiary care centres. The wide range in prevalence is partly explained by the absence of uniform pathological and clinical definitions. Histological findings form the basis of the diagnosis pulmonary hypoplasia, however complete autopsy data were often not available [2]. An international recognized definition of pulmonary hypoplasia is lacking, and it rather is a diagnosis by exclusion [6]. Congenital pneumonia, infant respiratory distress syndrome (IRDS) and pulmonary hypoplasia sometimes occur simultaneously, and have overlapping symptoms [1]. Moreover, there were methodological problems in the reviews, such as differences in follow-up and lack of blinded assessment of the endpoints.

Pregnancies complicated by midtrimester PPROM are associated with high immediate and long-term costs. These are caused by extended maternal hospital admissions, increased incidence of premature delivery, and frequent neonatal complications hereafter requiring NICU-admission.

Amnioinfusion might improve fetal outcome by preventing pulmonary hypoplasia, by preventing neurological complications, increasing time to delivery interval, and improving fetal biophysical profile through prevention of umbilical cord compression. It also might prevent fetal deformity [7]. Porat et al. [8] reviewed serial transabdominal amnioinfusion and meta-analysed observational as wel as randomised studies. The only two included randomised studies however were on women with PPROM between 24 and 34 weeks whereas the critical interval for development of pulmonary hypoplasia, the canalicular phase, exists between 16 and 28 weeks gestation. One quasi-randomised study included women with PPROM before 24 weeks. There were two observational studies on PPROM before 24 weeks. Recently, Roberts et al. [9] published the only randomized trial on PPROM between 16 and 24 weeks. They found no difference in the primary outcome (perinatal mortality 19/28 vs. 19/28; RR 1.0; 95% CI0.70-1.43), maternal or neonatal morbidity. The observed difference in long term outcome (4/28 morbidity free survival at 2 years in the treatment group vs 0/28 in the group with expectant management) justifies further study.

Adverse events after antepartum transabdominal amnioinfusion have been reported. It is not clear which adverse effects are to be attributed to the procedure rather than to the condition of PPROM and oligohydramnios, since conservatively managed PPROM carries high risks inherent to the condition itself [7]. Waters and Mercer recently reviewed perinatal mortality after conservatively managed PPROM <26 weeks [5]. Meta-analysis of 6 studies (n = 275) shows a perinatal mortality of 54%, (if restricted to <24 weeks this incidence is 57%). These data are biased by the fact that patients not amenable to continued expectant management were often excluded (i.e. stillbirths, pregnancy terminations), and therefore survival is likely to be overestimated.

A retrospective analysis by Van der Heijden et al. on outcome after PPROM in three tertiary centres in The Netherlands was recently published [10]. Their study included 14 multiple pregnancies. When these were excluded, in 164 singleton pregnancies with PROM before 24 weeks there was a perinatal mortality of 71% Of all mortality 38% occurred in the neonatal period (data not published). It can be questioned whether all pregnancies with PROM <24 weeks have been referred. Lethal pulmonary hypoplasia was documented in only 16 cases (10%), which is probably an underestimation as discussed before (personal communication by Van der Heijden).

In summary, midtrimester PPROM is associated with a high incidence of perinatal mortality, pulmonary hypoplasia and other neonatal complications. Exact incidences are difficult to obtain because of selection biases, probably leading to substantial underreporting. Diagnosis of pulmonary hypoplasia is difficult due to overlapping symptoms and absence of uniform definitions. Amnioinfusion might be beneficial, however there is no solid evidence to incorporate this seemingly safe procedure in daily practice. Therefore we believe there is the need to assess the role of amnioinfusion after midtrimester PPROM. We are currently conducting a multicentre randomised controlled clinical trial. This study is conducted within the Dutch Obstetric Consortium, a collaborative effort of obstetric clinics in The Netherlands to perform clinical trials. Seven Dutch perinatal centres with NICU facilities participate in this trial.

## Methods/Design

### Aims

The primary aim of this study is to evaluate the effectiveness of amnioinfusion compared to expectant management for relieving oligohydramnios in women with midtrimester PPROM occurring before 24 weeks gestational age in reducing perinatal mortality and neonatal morbidities.

### Participants/eligibility criteria

All women with a singleton pregnancy who are first diagnosed between 16 and 24 weeks gestational age with oligohydramnios secondary to PPROM, at least 72 hours after PPROM was diagnosed, but no longer than 21 days after the diagnosis of oligohydramnios, are eligible for the trial. Women with oligohydramnios secondary to iatrogenic PPROM are also eligible.

We will exclude women with signs of uterine contractions, (8 uterine contractions per hour) intrauterine infection (temperature > 38°C plus fetal tachycardia or uterine tenderness or foul/purulent amniotic fluid), a pregnancy complication (hypertension, HELLP syndrome, preeclampsia or other) in which there is a need for termination of pregnancy, placental or major structural fetal anomalies, signs of cervical incompetence (visible cervical dilatation or a cervical length of <25 mm), and women whose child has signs of fetal distress (abnormal biophysical profile).

### Procedures, recruitment, randomisation and collection of data

The research nurse and/or the staff of participating hospitals will identify eligible women. Prior to randomisation, in all patients amniotic fluid loss will be objectified by sterile speculum examination for visible fluid loss from the cervical os, by a nitrazine- and/or ferning test. Speculum examination will be performed to exclude signs of cervical incompetence (visible dilatation). Hereafter patients will undergo an ultrasound examination to determine the single deepest pocket (SDP) of amniotic fluid and to exclude placental and or fetal structural anomalies. At this time ultrasound measurements used in the prediction of pulmonary hypoplasia - TC/AC (thoracic circumference/abdominal circumference), TC/FL (thoracic circumference/femur length), Doppler measurement of pulmonary artery proximal branch peak systolic velocity, 3D lung volume measurement -, will be performed by specialized personnel.

**Figure 1:**
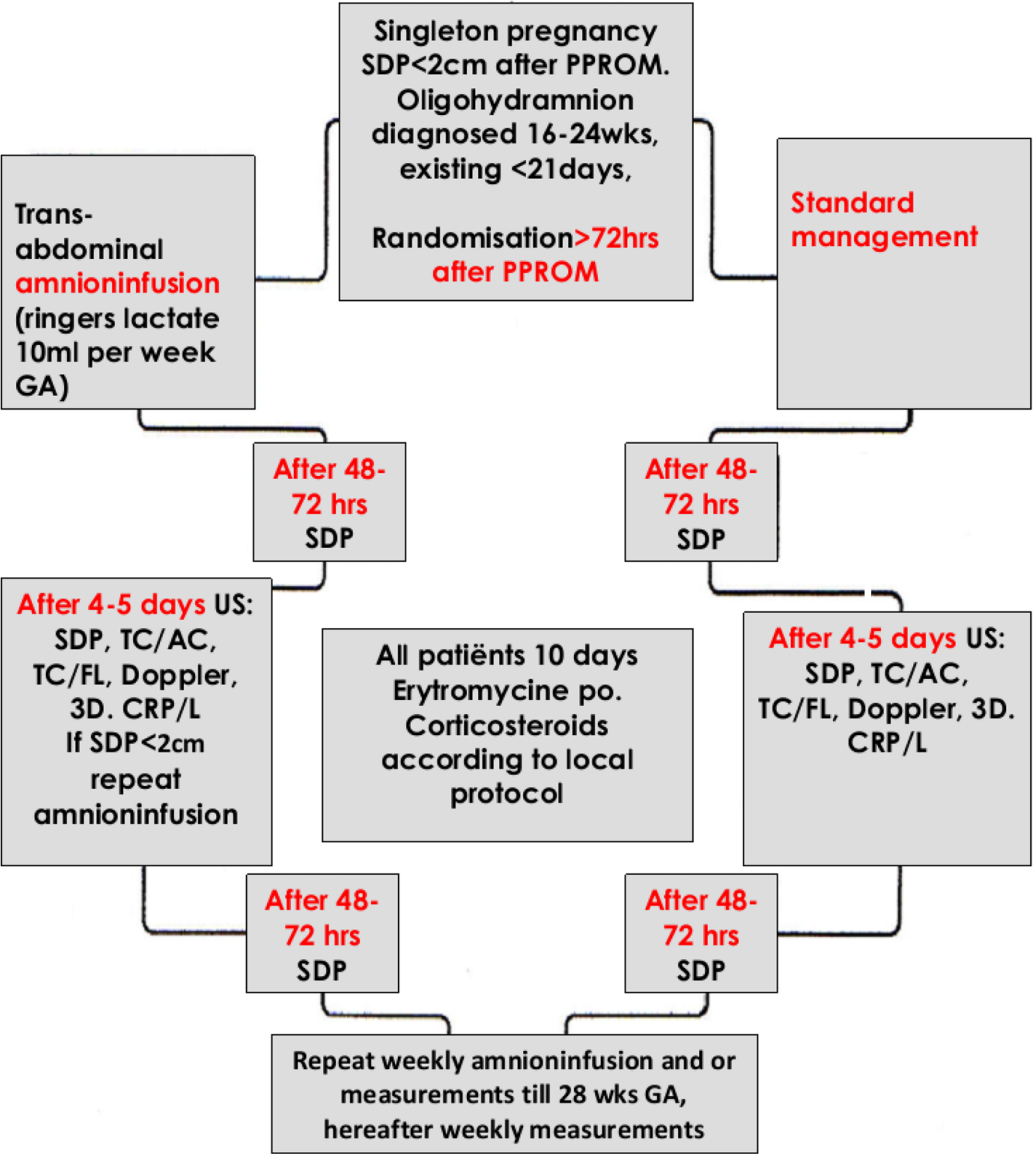
Flowchart PPROMEXIL-III trial.

If oligohydramnios is present (SDP < 2 cm) patients will be counselled for the study. Randomisation, will take place immediately after informed consent has been obtained, at least 72 hours after diagnosis of PPROM. See also Figure 1.

Randomisation will be performed, using an internet-based procedure, with the use of a permuted-block design, after informed consent and baseline data have been entered in a web-based database system.

Treatment allocation will obviously have to be unblinded for patients and clinicians and sonographers, and the personnel performing the ultrasound investigations, however, allocation will remain blinded for paediatricians, pathologists and/or radiologists who are assessing outcomes.

All data are collected, coded and processed with adequate precautions to ensure patient confidentiality. The investigators will publish the results of the study in a peer reviewed medical journal as soon as appropriate.

### Interventions

The intervention being evaluated is trans-abdominal amnioinfusion. After sterile preparation of the abdomen, a pocket of fluid is identified by ultrasound guidance after which a needle is advanced into this pocket. After insuring proper placement by withdrawing a small amount of fluid, the desired volume of fluid (Ringers lactate), defined by the number of weeks of gestational age times 10 millilitres, is infused manually. This procedure is repeated on a weekly basis if oligohydramnios re-occurs or persists.

If, after initial amnioinfusion, uterine contractions and signs of infection have been excluded, discharge is optional. Fluid retention will be assessed by measurement of SDP 48–72 hours after first amnioinfusion. Five to seven days after initial amnioinfusion, SDP is reassessed, as well as infection parameters (white cell count and CRP). Measurement of TC/AC, TC/FL, Doppler measurement, 3D lung volume measurement, will be performed by specialised personnel. If persisting or re-occurring oligohydramnios is diagnosed, amnioinfusion will be repeated. The same procedure will be repeated on a weekly basis until 28 weeks’ gestation.

Patients randomised to expectant management will undergo the same clinical and ultrasound examination bi-weekly (once a week measurement of SDP, TC/AC, TC/FL, Doppler, 3D lung volume, infection parameters, and on the second weekly visit assessment of fetal well-being and measurement of SDP). These patients can, if signs of premature labour or infection have been excluded, at the discretion of the local physician, be discharged as well (Figure 1). All discharged patients will be instructed to take their temperature twice daily and contact the hospital in case of fever (temperature > 37.8°C, rectally measured) or suspected infection. Patients with midtrimester PPROM are usually hospitalised after 24 weeks in a tertiary centre.

### Use of co-intervention

Corticosteroids are given according to local protocol, usage will be recorded in the case report form. All patients will receive treatment with antibiotics (Erythromycin orally 250 mg 4 times per day for ten days).

Patients may stop participating in the study at any time for any reason if they wish to do so without any consequences. The investigator can decide to withdraw a subject from the study for urgent medical reasons.

### Study parameters/endpoints

We will compare two groups:

1) Amnioinfusion for midtrimester PPROM with oligohydramnios, and

2) Expectant management for midtrimester PPROM with oligohydramnios.

The primary outcome measure will be perinatal mortality, defined as intrauterine death, intrapartum death or neonatal death in the first 28 days of life.

Secondary outcomes are:

• Gestational age at delivery.

• Time from membrane rupture to delivery.

• Indication for delivery.

• Successful amnioinfusion (defined as retention of infused fluid as defined by a single deepest pocket of amniotic fluid of more than 2 cm for at least 48 hours).

• Placental abruption.

• Cord prolapse.

• Chorioamnionitis, (defined as fever before or during labour as a temperature greater than 37.5°C on two occasion more than one hour apart or a temperature > 38.0°C with either uterine tenderness (or contractions), leucocytosis, maternal or fetal tachycardia, or a foul-smelling vaginal discharge in absence of any other cause of hyperpyrexia).

• Fetal trauma due to puncture.

• Maternal length of stay in hospital.

Occurrence of secondary outcomes placental abruption, cord prolapse, chorioamnionitis and fetal trauma will be related to the number of amnioinfusions that have been performed.

Neonatal endpoints:

• Lethal pulmonary hypoplasia diagnosed according to radiological, clinical and pathological criteria. Pathological criteria are based on radial alveolar counts and lung/body weight ratios according to Askenazi and Perlman [11], when radial alveolar counts cannot be obtained, criteria by Wigglesworth et al. will be used [12]. Clinical criteria used to diagnose lethal pulmonary hypoplasia are: immediate onset of severe respiratory insufficiency after birth, small lung capacity and requirement of high ventilatory pressures in the absence of obstruction or atelectasis. Radiological criteria according to Leonidas will be used [13].

• Non-lethal pulmonary hypoplasia diagnosed using same clinical and radiological criteria in surviving neonates.

• Survival till discharge from NICU.

• Neonatal mortality, defined as neonatal death in the first 28 days of life.

• Chronic lung disease (CLD), CLD defined as oxygen dependency at 28 days of life [14].

• Number of days on ventilatory support.

• Length of stay in hospital.

• Necrotising enterocolitis (NEC) more than stage I and defined according to the criteria of Bell et al. [15].

• Periventricular leukomalacia (PVL) more than grade I and defined according to the classification described by De Vries et al. [16].

• Severe intraventricular hemorrhage (IVH) more than grade II and defined according to the criteria of Papile et al. [17].

• Proven neonatal sepsis defined as (1) Positive blood culture taken at birth or (2) within 72 hours two or more symptoms of infection (apnea, temperature instability, lethargy, feeding, intolerance, respiratory distress, hemodynamic instability) plus one of three items: (a) positive blood culture (culture proven sepsis); (b) CRP > 20 (suspicion sepsis); (c) positive surface cultures of a known virulent pathogen (suspicion sepsis).

The neonatal endpoints will be defined by two neonatologists. The status of these endpoints will be evaluated at six months corrected age. Additional application for long-term follow-up of children (2 and 5 years) and mothers will be performed.

### Safety reporting

This study has been approved by the ethics committee (METC) of the Academic Medical Centre Amsterdam and by the boards of management of all participating hospitals.

In accordance with the National Medical Research Act (WMO, Section 10, Subsection 1), the investigator will inform the subjects and the reviewing accredited METC in case it appears that the disadvantages of participation may be significantly greater than was foreseen in the research proposal. All observed or volunteered adverse events, regardless of suspected causal relationship to the intervention, will be recorded.

An adverse event (AE) is defined as an event after which the intervention has to be stopped. Reasons for discontinuation are placental abruption, cord prolapse, chorioamnionitis, fetal loss, fetal trauma due to puncture, premature labour and delivery.

A serious adverse event (SAE) is defined as fetal or maternal death or illness necessitating IC or CCU treatment. All SAEs will be reported to the accredited METC that approved the protocol, according to the requirements of that METC. In addition to the expedited reporting of SAE’s, the principal investigator will submit, once a year throughout the clinical trial, a safety report to the accredited METC.

A Data Safety Monitoring Board (DSMB) will be established prior to start of the trial.

### Sample size calculation

A sample size calculation was based on an expected rate of perinatal mortality of 70% with expectant management, to be reduced to 35% with amnioinfusion. Using a two-sided test, with a β-error of 0.20 and an α-error of 0.05 a sample size of 56 women (28 in each arm) is needed.

An interim analysis is planned after the follow up data of the first 28 women that have been included. The analysis will be performed using the O’Brien-Flemming alpha spending function meaning a nominal P value of less than 0.005 will be considered to indicate statistical significance [16]. If there is a significant difference in the primary outcome the trial will be stopped.

### Statistical analysis

The analysis of the randomised clinical trial will be performed on an intention-to-treat principle. The differences between the amnioinfusion and expectant management will be assessed by calculating the ratio of the outcome rates in the two groups. Hence, the measure of association is a relative risk (RR) with a 95% confidence interval (CI), calculated using a log-binomial model. Time to delivery will be evaluated by Cox proportional hazard analysis, Kaplan-Meier estimates and tested with a log rank test.

In case of equivalence between outcomes, the analysis will be repeated on an “as-treated” basis. Subsequently, primary and secondary outcomes will be described in a subgroup of women with successful amnioinfusion (retention of infused fluid first 48 hours). All analyses will be adjusted for the fact that an interim analysis will be performed using the O’Brien-Flemming alpha spending function [18]. Consequently, a nominal P value of less than 0.049 will be considered to indicate statistical significance.

## Discussion

There are signs that pregnancies complicated by oligohydramnios after midtrimester PPROM might benefit from amnioinfusion. However, currently there is insufficient evidence to recommend this procedure. The benefits might be increased neonatal survival and decreased pulmonary complications, especially pulmonary hypoplasia. Potential harms include placental abruption, premature labour and delivery, cord prolapse, chorioamnionitis, fetal loss, fetal trauma due to puncture. Of these, only the last mentioned is not a known potential complication of the underlying condition itself as well (midtrimester PPROM). Expectant management indeed carries these same risks. At present, there is no evidence on which a rational choice between expectant management or therapeutic amnioifusion can be based.

## Abbreviations

3D: 3-dimensional; AC: Abdominal circumference; AE: Adverse event; BPD: Bronchopulmonary dysplasia; CCU: Cardiac care unit; CLD: Chronic lung disease; CRP: C-reactive protein; CTG: Cardiotocography; DSMB: Data Safety Monitoring Board; FL: Femur length; GA: Gestational age; IRDS: Infant respiratory distress syndrome; IVH: Intraventricular haemorrhage; METC: Medical research ethics committee (MREC); in Dutch: medisch ethische toetsingscommissie (METC); NEC: Necrotizing enterocolitis; NICU: Neonatal Intensive Care Unit; PPROM: Preterm prelabour rupture of membranes; PVL: Periventricular leucomalacia; SAE: Serious adverse event; SDP: Single deepest pocket (amniotic fluid); TC: Thoracic circumference; US: Ultrasound; WMO: Medical Research Involving Human Subjects Act (Wet Medisch-wetenschappelijk Onderzoek met Mensen).

## Competing interests

The authors declare that they have no competing interests.

## Authors’ contributions

ASPvT, DPvdH, EP, CW, JGN, SvK, ES, ALMM and BWM are member of the PPROMEXIL study group and were involved in conception and design of the study. ASPvT, drafted the manuscript. EP, MWMdL, CW, SAL, AM, MF, DO, FARJ, MW, MNB, CB and MP are local investigators at the participating centres and discussed and fine-tuned the final design of the study. All authors edited the manuscript and read and approved the final draft.

## Pre-publication history

The pre-publication history for this paper can be accessed here:

http://www.biomedcentral.com/1471-2393/14/128/prepub

## References

[B1] Kilbride HW Thibeault DW Neonatal complications of preterm rupture of membranes Clin Perinatol 2001 28 4 761 785 10.1016/S0095-5108(03)00076-9 11817188

[B2] Sherer DM Davis JM Woods JR Jr Pulmonary hypoplasia: a review Obstetr Gynecol Surv 1990 45 11 792 803 10.1097/00006254-199011000-00026 2234705

[B3] Laudy JA Wladimiroff JW The fetal lung. 2: Pulmonary hypoplasia Ultrasound Obstet Gynecol 2000 16 5 482 494 10.1046/j.1469-0705.2000.00252.x 11169336

[B4] Grisaru-Granovsky S Eitan R Kaplan M Samueloff A Expectant management of midtrimester premature rupture of membranes: a plea for limits J Perinatol 2003 23 3 235 239 10.1038/sj.jp.7210880 12732862

[B5] Waters TP Mercer BM The management of preterm premature rupture of the membranes near the limit of fetal viability Am J Obstet Gynecol 2009 201 3 230 240 10.1016/j.ajog.2009.06.049 19733274

[B6] Geary C Whitsett J Inhaled nitric oxide for oligohydramnios-induced pulmonary hypoplasia: a report of two cases and review of the literature J Perinatol 2002 22 1 82 85 10.1038/sj.jp.7210580 11840249

[B7] Gramellini D Fieni S Kaihura C Piantelli G Verrotti C Antepartum amnioinfusion: a review J Matern Fetal Neonatal Med 2003 14 5 291 296 10.1080/jmf.14.5.291.296 14986801

[B8] Porat S Amsalem H Shah PS Murphy KE Transabdominal amnioinfusion for preterm premature rupture of membranes: a systematic review and metaanalysis of randomized and observational studies Am J Obstet Gynecol 2012 207 5 393 e1-11 22999157 10.1016/j.ajog.2012.08.003

[B9] Roberts D Vause S Martin W Green P Walkinshaw S Bricker L Beardsmore C Shaw N McKay A Skotny G Williamson P Alfirevic Z Amnioinfusion in very early preterm premature rupture of membranes - pregnancy, neonatal and maternal outcomes in the AMIPROM randomised controlled pilot study Ultrasound Obstet Gynecol 2013 Epub 2013/11/23 10.1002/uog.13258 24265189

[B10] van der Heyden JL van der Ham DP van Kuijk S Notten KJ Janssen T Nijhuis JG Willekes C Porath M van der Post JA Halbertsma F Mol BW Pajkrt E Outcome of pregnancies with preterm prelabor rupture of membranes before 27 weeks’ gestation: a retrospective cohort study Eur J Obstet Gynecol Reprod Biol 2013 170 1 125 130 10.1016/j.ejogrb.2013.06.012 23845169

[B11] Askenazi SS Perlman M Pulmonary hypoplasia: lung weight and radial alveolar count as criteria of diagnosis Arch Dis Child 1979 54 614 618 10.1136/adc.54.8.614 507916 PMC1545796

[B12] Wigglesworth JS Desai R Use of DNA estimation for growth assessment in normal and hypoplastic fetal lungs Arch Dis Child 1981 56 601 605 10.1136/adc.56.8.601 7271300 PMC1627277

[B13] Leonidas JC Bhan I Beatty EC Radiographic chest contour and pulmonary air leaks in oligohydramnios-related pulmonary hypoplasia (Potter’s syndrome) Invest Radiol 1982 17 6 10 10.1097/00004424-198201000-00002 7076436

[B14] Shennan AT Dunn MS Ohlsson A Lennox K Hoskins EM Abnormal pulmonary outcomes in premature infants: prediction from oxygen requirement in the neonatal period Pediatrics 1988 82 4 527 532 3174313

[B15] Bell MJ Ternberg JL Feigin RD Keating JP Marshall R Barton L Brotherton T Neonatal necrotizing enterocolitis. Therapeutic decisions based upon clinical staging Ann Surg 1978 187 1 1 7 10.1097/00000658-197801000-00001 413500 PMC1396409

[B16] de Vries LS Eken P Dubowitz LM The spectrum of leukomalacia using cranial ultrasound Behav Brain Res 1992 49 1 1 6 10.1016/S0166-4328(05)80189-5 1388792

[B17] Papile LA Burstein J Burstein R Koffler H Incidence and evolution of subependymal and intraventricular hemorrhage: a study of infants with birth weights less than 1,500 gm J Pediatr 1978 92 4 529 534 10.1016/S0022-3476(78)80282-0 305471

[B18] O’Brien PC Fleming TR A multiple testing procedure for clinical trials Biometrics 1979 35 3 549 556 10.2307/2530245 497341

